# Supplementary material for: A Humanized Yeast Phenomic Model of Deoxycytidine Kinase to Predict Genetic Buffering of Nucleoside Analog Cytotoxicity
Source: Genes (Basel). 2019 Sep 30;10(10):770. doi: 10.3390/genes10100770 (PMC6826991; doi:10.3390/genes10100770)

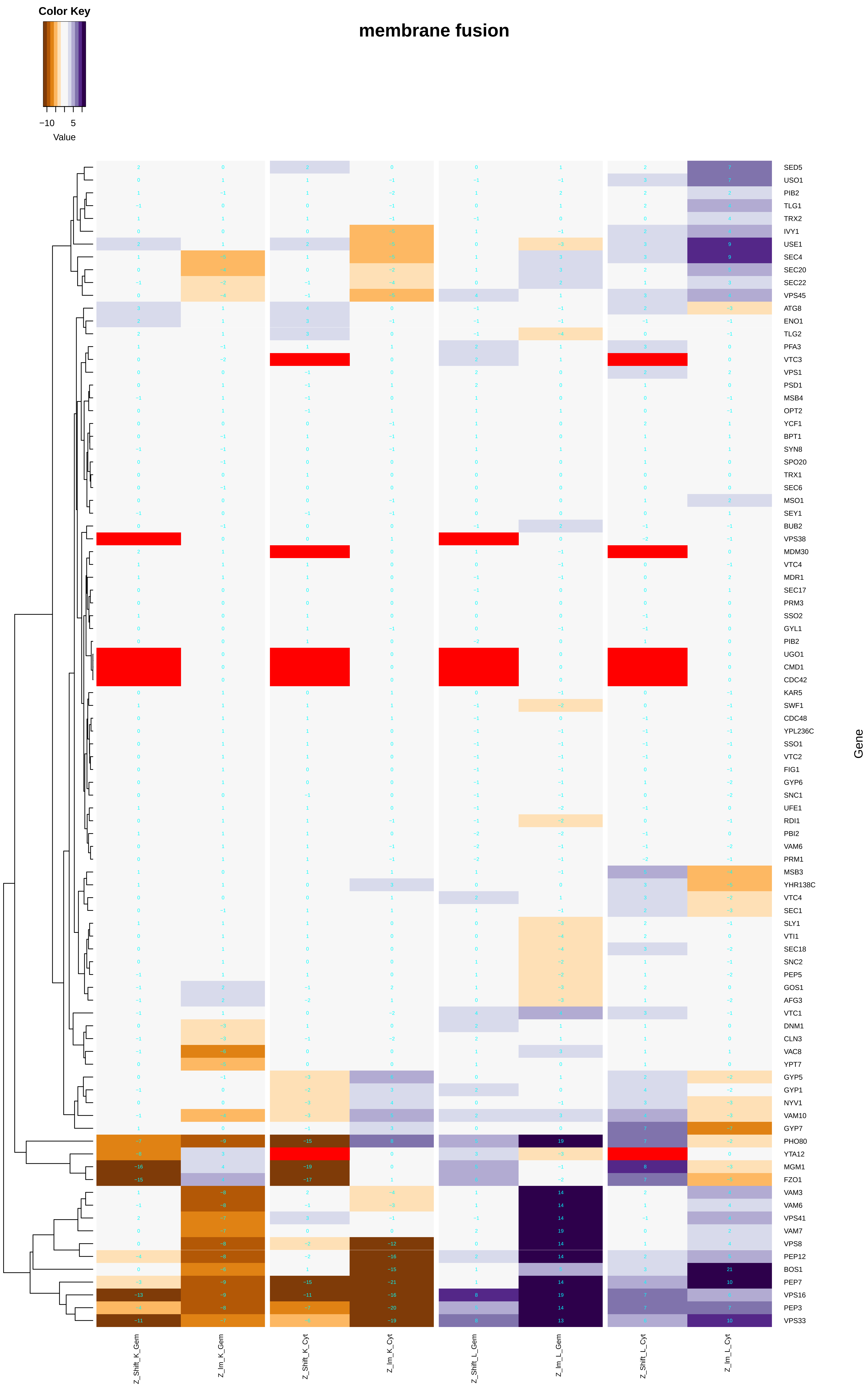
membrane fusion
Color Key
−10
5
Value
SED5
USO1
PIB2
TLG1
TRX2
IVY1
USE1
SEC4
SEC20
SEC22
VPS45
ATG8
ENO1
TLG2
PFA3
VTC3
VPS1
PSD1
MSB4
OPT2
YCF1
BPT1
SYN8
SPO20
TRX1
SEC6
MSO1
SEY1
BUB2
VPS38
MDM30
VTC4
MDR1
SEC17
PRM3
SSO2
GYL1
PIB2
UGO1
CMD1
CDC42
KAR5
SWF1
CDC48
YPL236C
SSO1
VTC2
FIG1
GYP6
SNC1
UFE1
RDI1
PBI2
VAM6
PRM1
MSB3
YHR138C
VTC4
SEC1
SLY1
VTI1
SEC18
SNC2
PEP5
GOS1
AFG3
VTC1
DNM1
CLN3
VAC8
YPT7
GYP5
GYP1
NYV1
VAM10
GYP7
PHO80
YTA12
MGM1
FZO1
VAM3
VAM6
VPS41
VAM7
VPS8
PEP12
BOS1
PEP7
VPS16
PEP3
VPS33
Gene
Z_Shift_K_Gem
Z_Im_K_Gem
Z_Shift_K_Cyt
Z_Im_K_Cyt
Z_Shift_L_Gem
Z_Im_L_Gem
Z_Shift_L_Cyt
Z_Im_L_Cyt

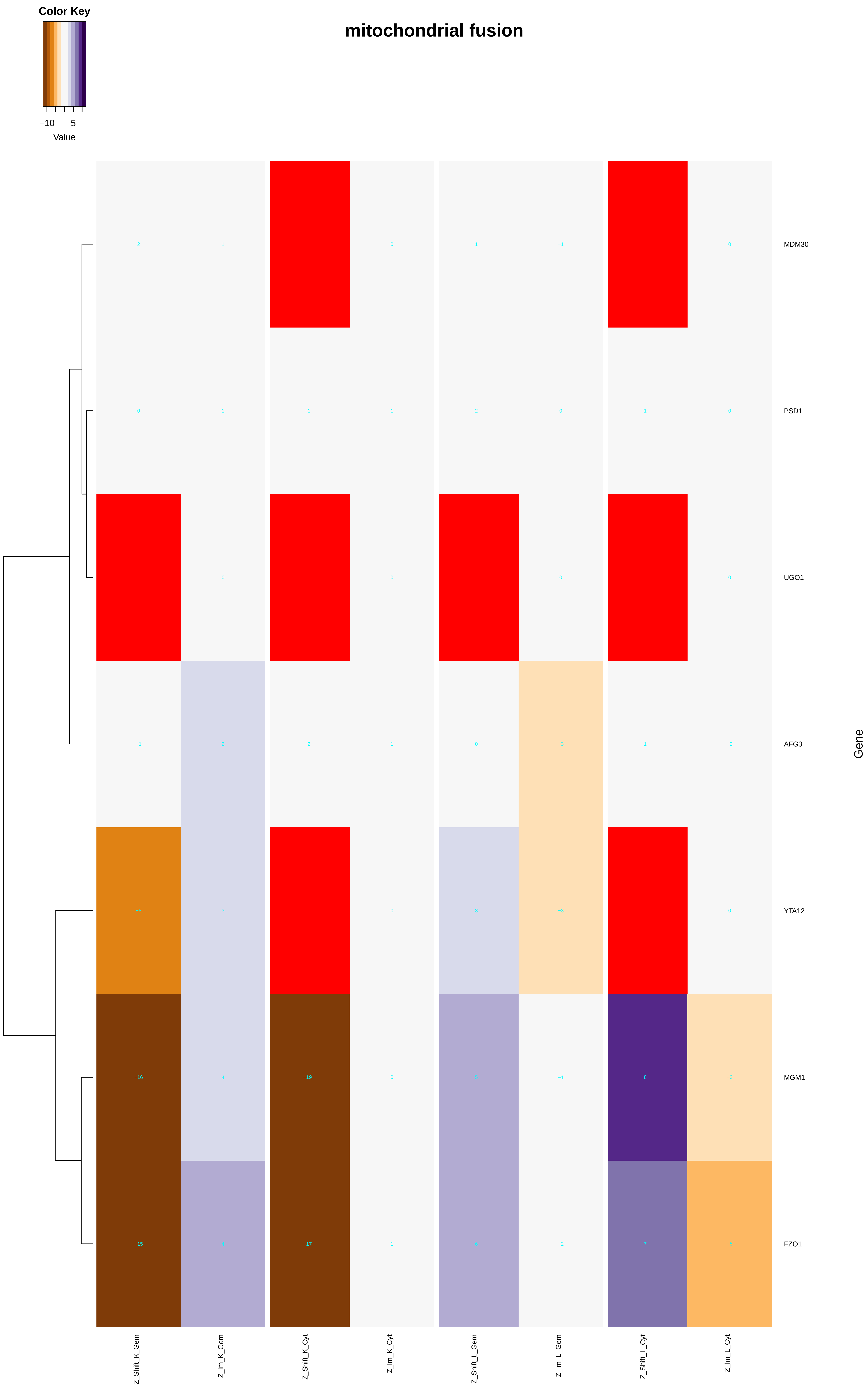

Color Key
−10
5
Value
mitochondrial fusion
MDM30
PSD1
UGO1
AFG3
YTA12
MGM1
FZO1
Gene
Z_Shift_K_Gem
Z_Im_K_Gem
Z_Shift_K_Cyt
Z_Im_K_Cyt
Z_Shift_L_Gem
Z_Im_L_Gem
Z_Shift_L_Cyt
Z_Im_L_Cyt

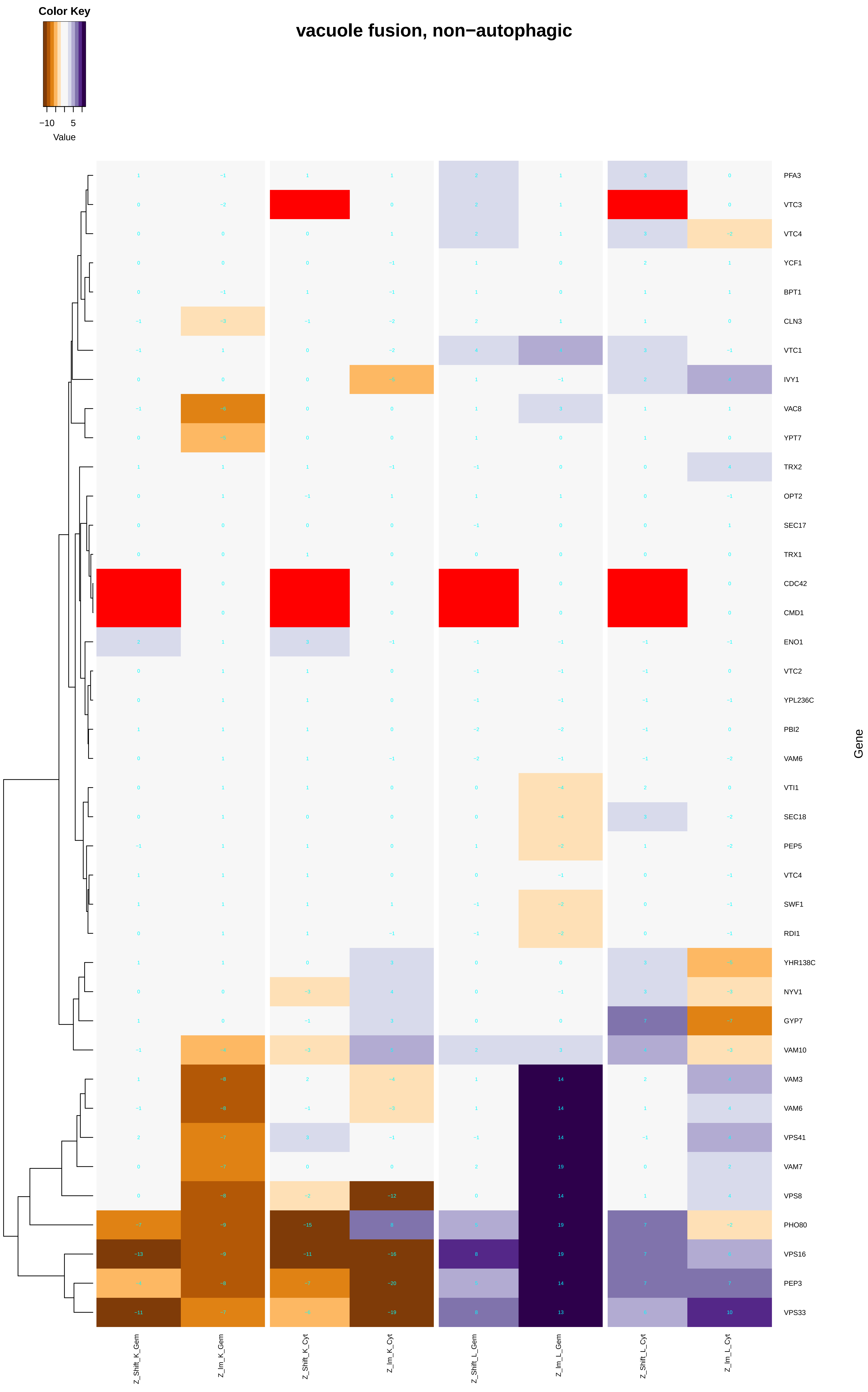

vacuole fusion, non−autophagic
Color Key
−10
5
Value
PFA3
VTC3
VTC4
YCF1
BPT1
CLN3
VTC1
IVY1
VAC8
YPT7
TRX2
OPT2
SEC17
TRX1
CDC42
CMD1
ENO1
VTC2
YPL236C
PBI2
VAM6
VTI1
SEC18
PEP5
VTC4
SWF1
RDI1
YHR138C
NYV1
GYP7
VAM10
VAM3
VAM6
VPS41
VAM7
VPS8
PHO80
VPS16
PEP3
VPS33
Gene
Z_Shift_K_Gem
Z_Im_K_Gem
Z_Shift_K_Cyt
Z_Im_K_Cyt
Z_Shift_L_Gem
Z_Im_L_Gem
Z_Shift_L_Cyt
Z_Im_L_Cyt

organelle membrane fusion

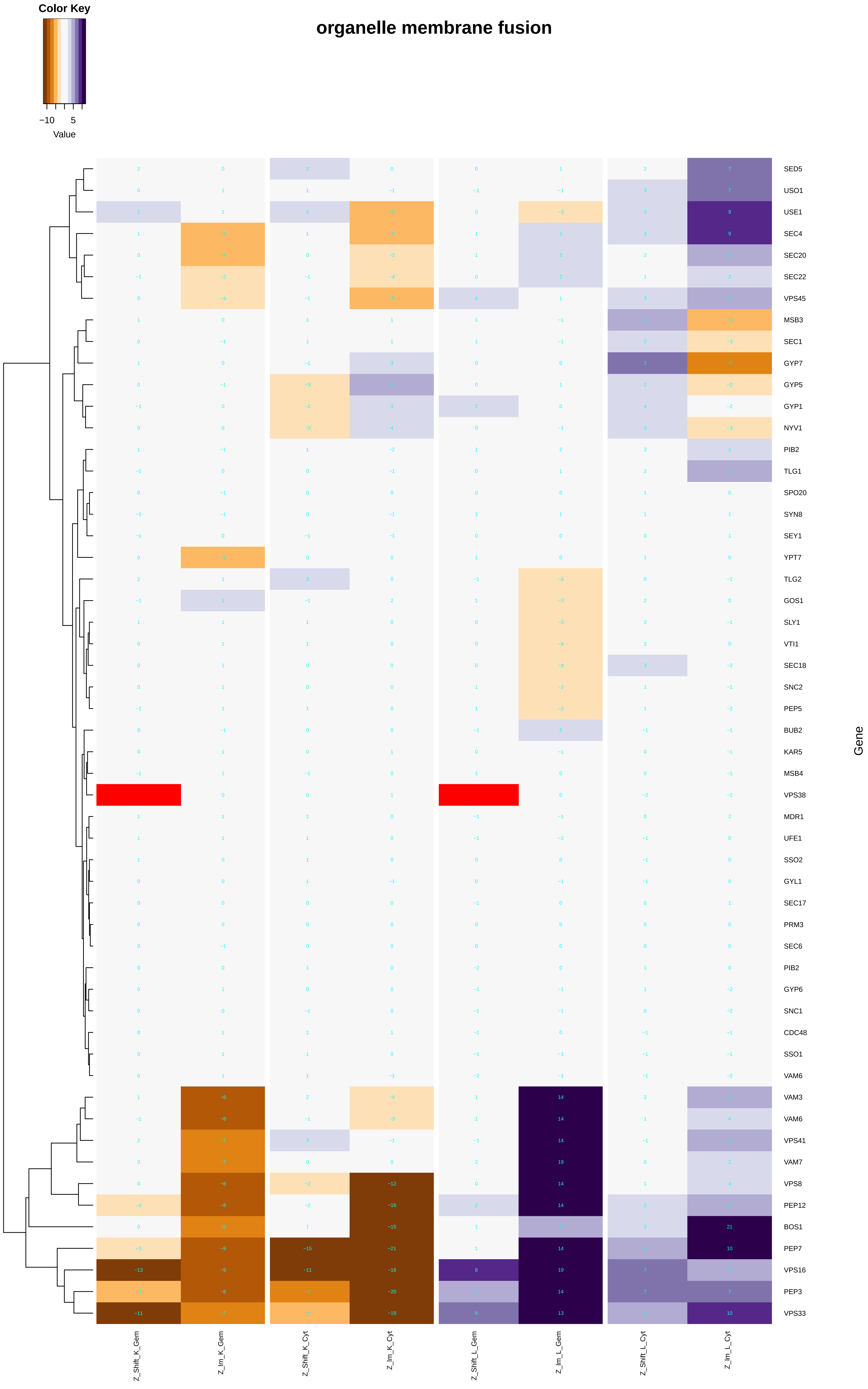

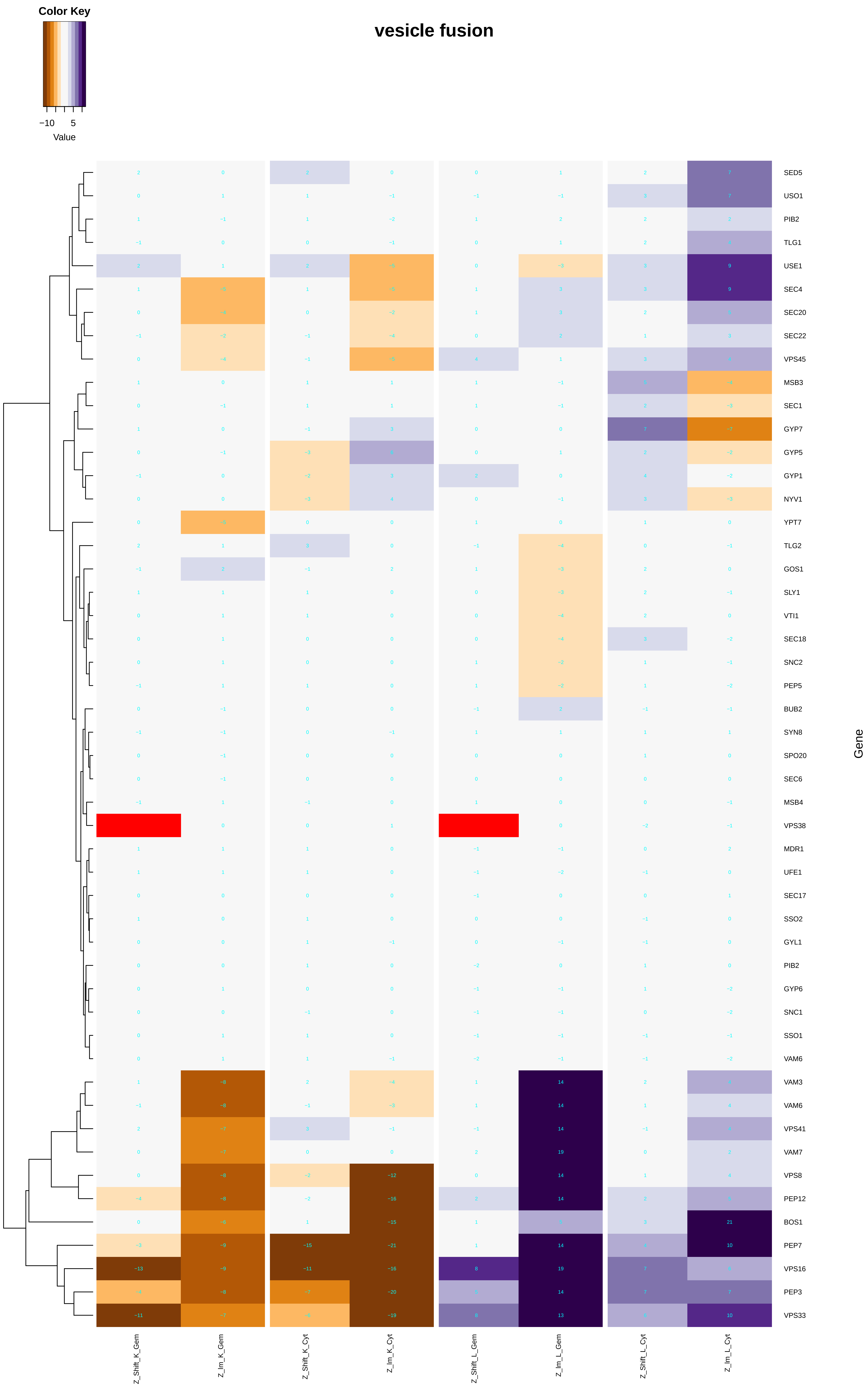
Color Key
−10
5
Value
vesicle fusion
SED5
USO1
PIB2
TLG1
USE1
SEC4
SEC20
SEC22
VPS45
MSB3
SEC1
GYP7
GYP5
GYP1
NYV1
YPT7
TLG2
GOS1
SLY1
VTI1
SEC18
SNC2
PEP5
BUB2
SYN8
SPO20
SEC6
MSB4
VPS38
MDR1
UFE1
SEC17
SSO2
GYL1
PIB2
GYP6
SNC1
SSO1
VAM6
VAM3
VAM6
VPS41
VAM7
VPS8
PEP12
BOS1
PEP7
VPS16
PEP3
VPS33
Gene
Z_Shift_K_Gem
Z_Im_K_Gem
Z_Shift_K_Cyt
Z_Im_K_Cyt
Z_Shift_L_Gem
Z_Im_L_Gem
Z_Shift_L_Cyt
Z_Im_L_Cyt

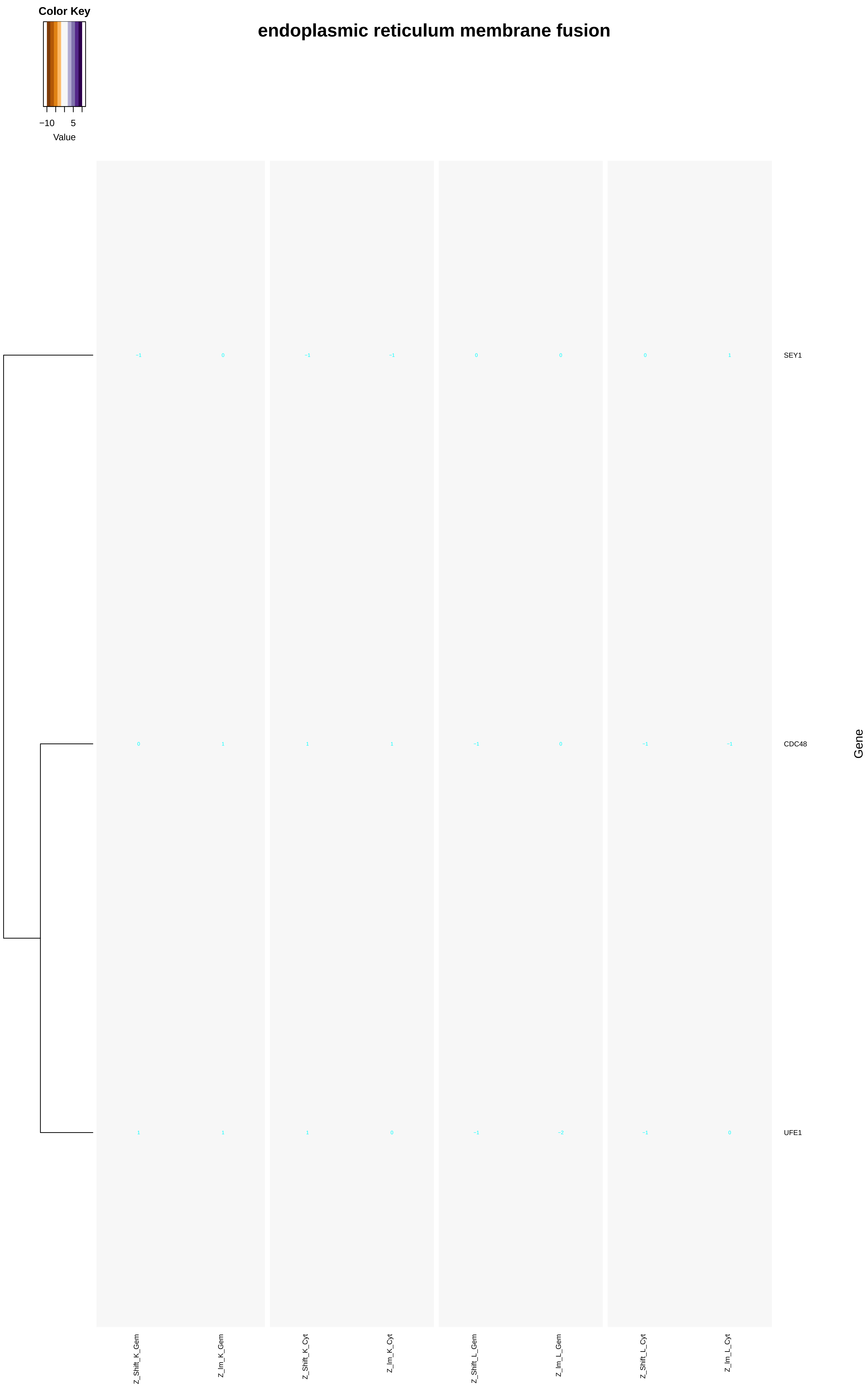
Color Key
−10
5
Value
endoplasmic reticulum membrane fusion
SEY1
CDC48
UFE1
Gene
−1 0 −1 −1 0 0 0 1
0 1 1 1 −1 0 −1 −1
1 1 1 0 −1 −2 −1 0
Z_Shift_K_Gem
Z_Im_K_Gem
Z_Shift_K_Cyt
Z_Im_K_Cyt
Z_Shift_L_Gem
Z_Im_L_Gem
Z_Shift_L_Cyt
Z_Im_L_Cyt

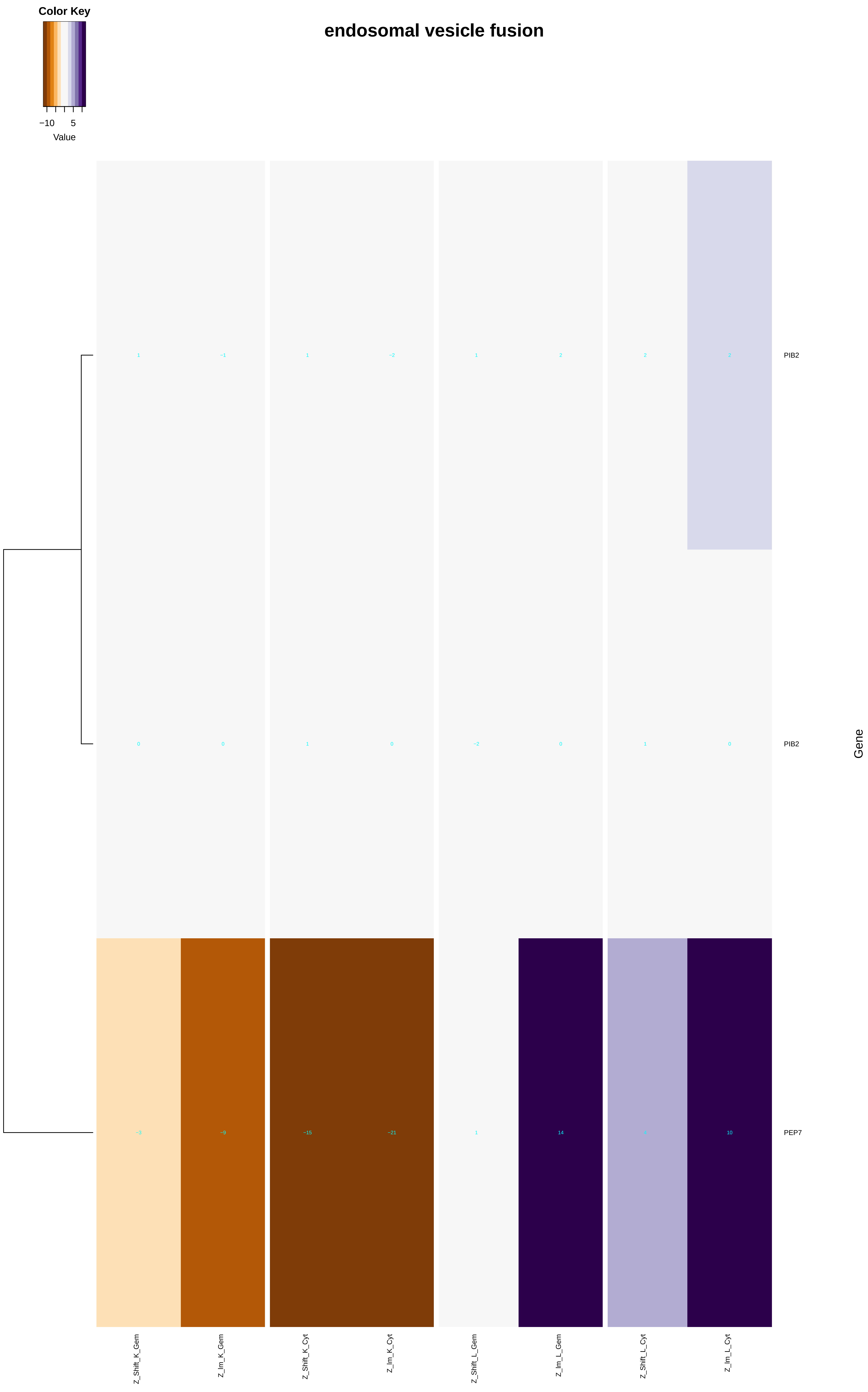

Color Key
−10
5
Value
endosomal vesicle fusion
PIB2
PIB2
PEP7
Gene
Z_Shift_K_Gem
Z_Im_K_Gem
Z_Shift_K_Cyt
Z_Im_K_Cyt
Z_Shift_L_Gem
Z_Im_L_Gem
Z_Shift_L_Cyt
Z_Im_L_Cyt

vesicle fusion with endoplasmic reticulum

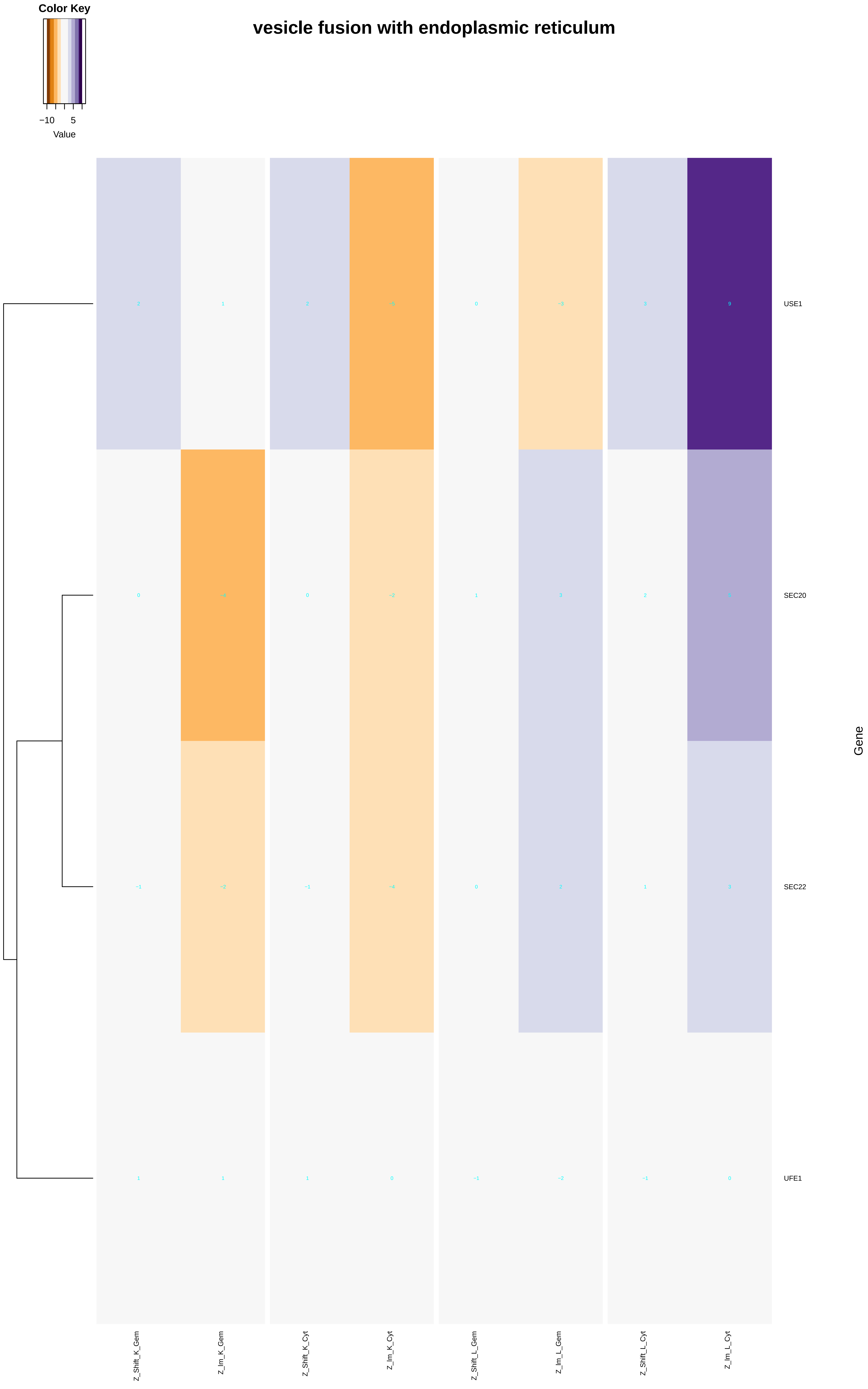

vesicle fusion with Golgi apparatus

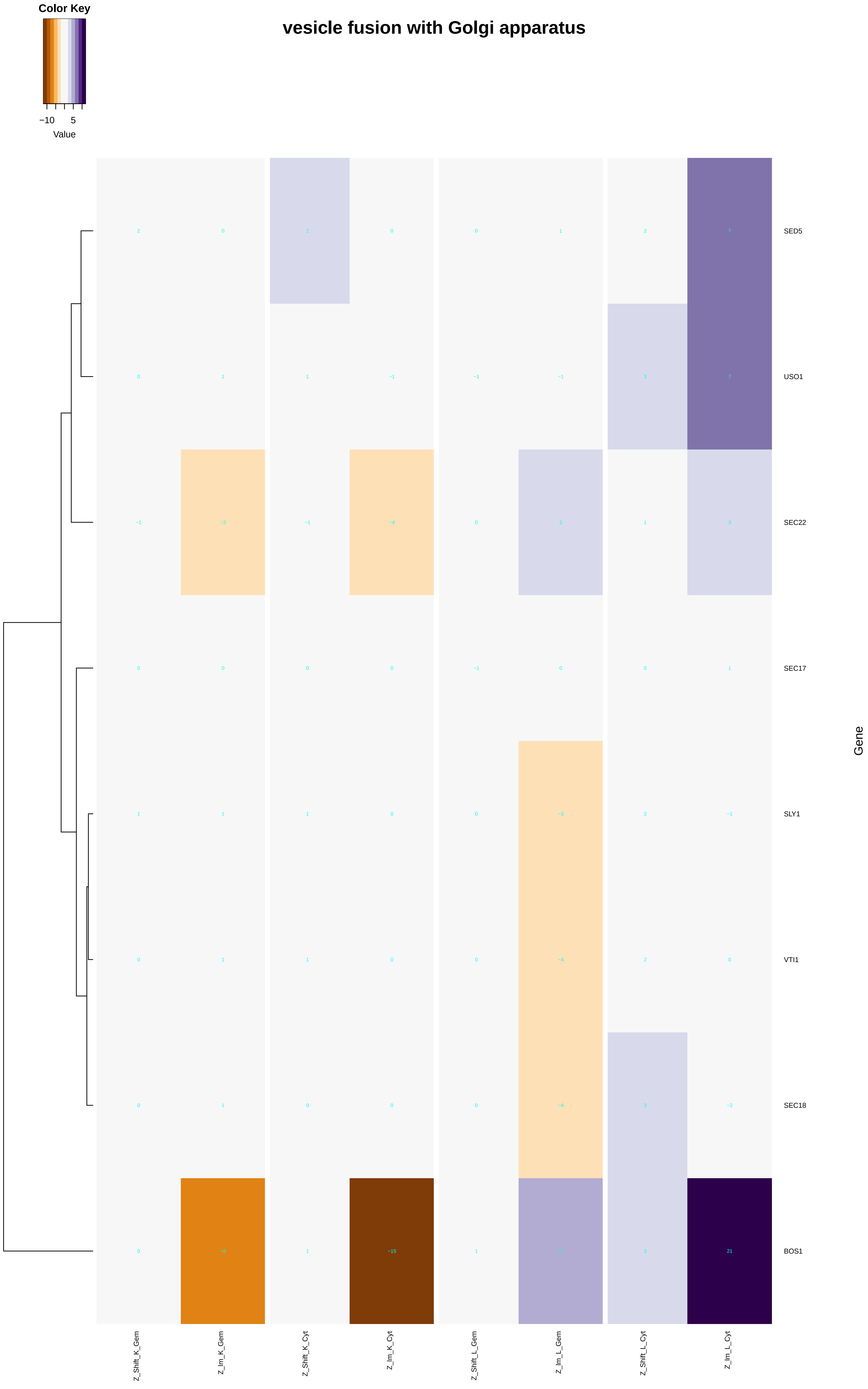

Supplement: Supplementary file 1 [file genes-10-00770-s001.zip › Additional_Files/Additional_File7_GOTermHeatmaps.zip!/Additional_File7_GOTermHeatmaps/membrane_fusion.pdf]
